# H1N1-associated hemophagocytic lymphohistiocytosis in a child with Down syndrome: A clinical challenge

**DOI:** 10.5339/qmj.2025.120

**Published:** 2025-12-14

**Authors:** Hamza Khoursheed, Aref Dawoud, Ahmad Qandeel, Hani Qteishat, Suleiman Sweedan

**Affiliations:** 1 Jordan University of Science and Technology, Irbid, Jordan *Email: hamzeh_2002@hotmail.com

**Keywords:** Hemophagocytic lymphohistiocytosis, Down syndrome, H1N1 influenza, secondary HLH, immunosuppression therapy

## Abstract

**Background:**

Hemophagocytic lymphohistiocytosis (HLH) is a rare, life-threatening hyperinflammatory syndrome characterized by excessive immune activation. Children with Down syndrome (DS) (trisomy 21) are at increased risk of severe infections and immune dysregulation. Although viral infections are known triggers of secondary HLH, H1N1 influenza is an uncommon cause in this population.

**Case presentation:** We report a 1.8-month-old male infant with DS and multiple comorbidities—including gastroesophageal reflux disease (GERD), recurrent aspiration pneumonia, and seizures—who presented with fever, hypoxemia, and splenomegaly. Laboratory investigations revealed cytopenias, hyperferritinemia (>1,650 ng/mL), and hypofibrinogenemia, fulfilling five of the HLH-2004 diagnostic criteria. H1N1 influenza was confirmed as the infectious trigger. The patient was treated with standard-dose dexamethasone and etoposide, along with oseltamivir. Despite concerns about treatment-related toxicity in DS, he tolerated the regimen without hematologic or infectious complications. He achieved clinical remission with normalization of inflammatory markers and hematologic parameters. At discharge and at three-month follow-up, he remained well with no recurrence or neurological sequelae.

**Discussion:** This case highlights the diagnostic and therapeutic challenges of managing HLH in children with DS. The use of conventional HLH therapy, including etoposide, was effective and well-tolerated despite theoretical concerns in this population. Although central nervous system symptoms were present, they resolved with treatment. H1N1 influenza should be considered a potential viral trigger in immunocompromised children presenting with HLH-like features.

**Conclusion:**

H1N1 influenza-induced HLH is exceptionally rare in infants with DS. The positive outcome in this case supports the safe use of standard HLH protocols in this vulnerable population and highlights the importance of early diagnosis and multidisciplinary management to optimize outcomes.

## 1. INTRODUCTION

Down syndrome (DS), caused by trisomy 21, results from the presence of an additional copy of human chromosome 21 and is the most prevalent chromosomal anomaly, affecting approximately 1 in 800 live births worldwide.^1^ It is well established that trisomy 21 is associated with immune dysfunction, affecting both innate and adaptive immune responses.^2^ Hemophagocytic lymphohistiocytosis (HLH) is a severe hyperinflammatory syndrome characterized by uncontrolled immune activation, driven primarily by excessive macrophage activation and the overproduction of proinflammatory cytokines.^3^ HLH can be classified as either primary (familial) HLH, resulting from genetic mutations that impair immune regulation, or secondary HLH, which arises in response to triggers such as infections, malignancies, autoimmune diseases, chronic inflammation, or certain immunomodulatory therapies, including immune checkpoint inhibitors.^4^ Among infectious causes, viral pathogens are the most frequent triggers.^4^ The clinical presentation of HLH is often nonspecific, frequently mimicking severe systemic infections. Patients typically exhibit fever, hepatosplenomegaly, and multiorgan involvement, along with characteristic laboratory abnormalities, such as pancytopenia, elevated liver transaminases, hyperbilirubinemia, coagulopathy, hypofibrinogenemia, hypoalbuminemia, hyponatremia, hypertriglyceridemia, and hyperferritinemia. Here, we present a rare case of secondary HLH triggered by H1N1 influenza in an infant with trisomy 21, highlighting the diagnostic challenges and complexities of managing HLH in pediatric patients with DS. Written informed consent was obtained from the patient’s legal guardians for the publication of this case report. Institutional approval for publication was granted by the hospital’s ethics committee (IRB Approval No.: F2025/689) in accordance with its case reporting policy.

## 2. CASE PRESENTATION

A 1.8-month-old male infant with DS was brought by his parents to the Emergency Department (ED) on December 12, 2024, at a tertiary care public teaching hospital in Jordan. He presented with tachypnea, hypoxemia (oxygen saturation of 90%), and altered consciousness.

He was born at 38 weeks via spontaneous vaginal delivery after an unremarkable antenatal course, with no reported maternal infections or pregnancy complications. His past medical history included asthma, gastroesophageal reflux disease (GERD), seizures, and recurrent aspiration pneumonia. Two weeks prior to the current presentation, he had been hospitalized for oxygen desaturation (83–85%) accompanied by low-grade fever and upper respiratory symptoms. At that time, both H1N1 and COVID-19 polymerase chain reaction (PCR) tests were negative. He received supportive care and was discharged after one week with home oxygen therapy (0.5 L/min via nasal cannula) due to persistent hypoxemia and chronic pulmonary issues. He was also placed on home pulse oximetry monitoring as part of his follow-up plan. On the morning of the current presentation, his mother noticed coughing and rhinorrhea, and despite increasing oxygen flow to 2 L/min, his oxygen saturation remained at 90%. On examination in the ED, notable findings included cough, increased work of breathing, hypoxemia, and splenomegaly.

Based on the tests performed during his Emergency Department assessment (Table 1), the patient fulfilled five of the eight HLH-2004 diagnostic criteria, confirming secondary HLH. He presented with persistent fever—one of the hallmark features of HLH—in the context of severe hypoxemia and respiratory distress. Splenomegaly observed on examination further supported the diagnosis. Laboratory investigations revealed pancytopenia, with significant reductions in platelets (34×10^9^/L), white blood cell count (3.96×10^3^/mm^3^), and absolute neutrophil count (ANC: 130 cells/μL), consistent with the characteristic cytopenias observed in HLH. The patient also exhibited hyperferritinemia (>1,650 ng/mL), hypofibrinogenemia (101 mg/dL), and elevated lactate dehydrogenase (LDH: 739 U/L), reflecting the excessive inflammatory response and underlying hemophagocytosis. Given the presence of an identifiable viral trigger (H1N1 influenza) in a patient with DS—a population known to exhibit immune dysregulation—the clinical and laboratory findings strongly supported infection-driven HLH, necessitating urgent immunosuppressive therapy.

Upon confirmation of HLH, whole-exome sequencing was performed to evaluate HLH-related mutations and primary immunodeficiencies; the results were negative, supporting the diagnosis of secondary HLH. The patient was promptly initiated on corticosteroids and etoposide in accordance with the HLH-2004 treatment protocol. Dexamethasone (10 mg/ml) was administered to suppress the hyperinflammatory response, reduce cytokine overproduction, and control the immune dysregulation characteristic of HLH. The decision to use etoposide, a key component of HLH-directed therapy, was carefully considered due to concerns regarding potential myelosuppression.^5^ However, given the severity of the hyperinflammatory state and the risk of multiorgan failure, etoposide was introduced at standard dosing (150 mg/m^2^ intravenously once weekly for eight weeks) and was well tolerated, with no significant hematologic toxicity or infectious complications. Following initial evaluation and stabilization in the Emergency Department, the patient was admitted to the pediatric intensive care unit (PICU) for continuous monitoring and further management of suspected HLH and respiratory distress. He demonstrated progressive clinical improvement, including resolution of fever, stabilization of blood counts, and a decline in inflammatory markers.

**Figure 1:**
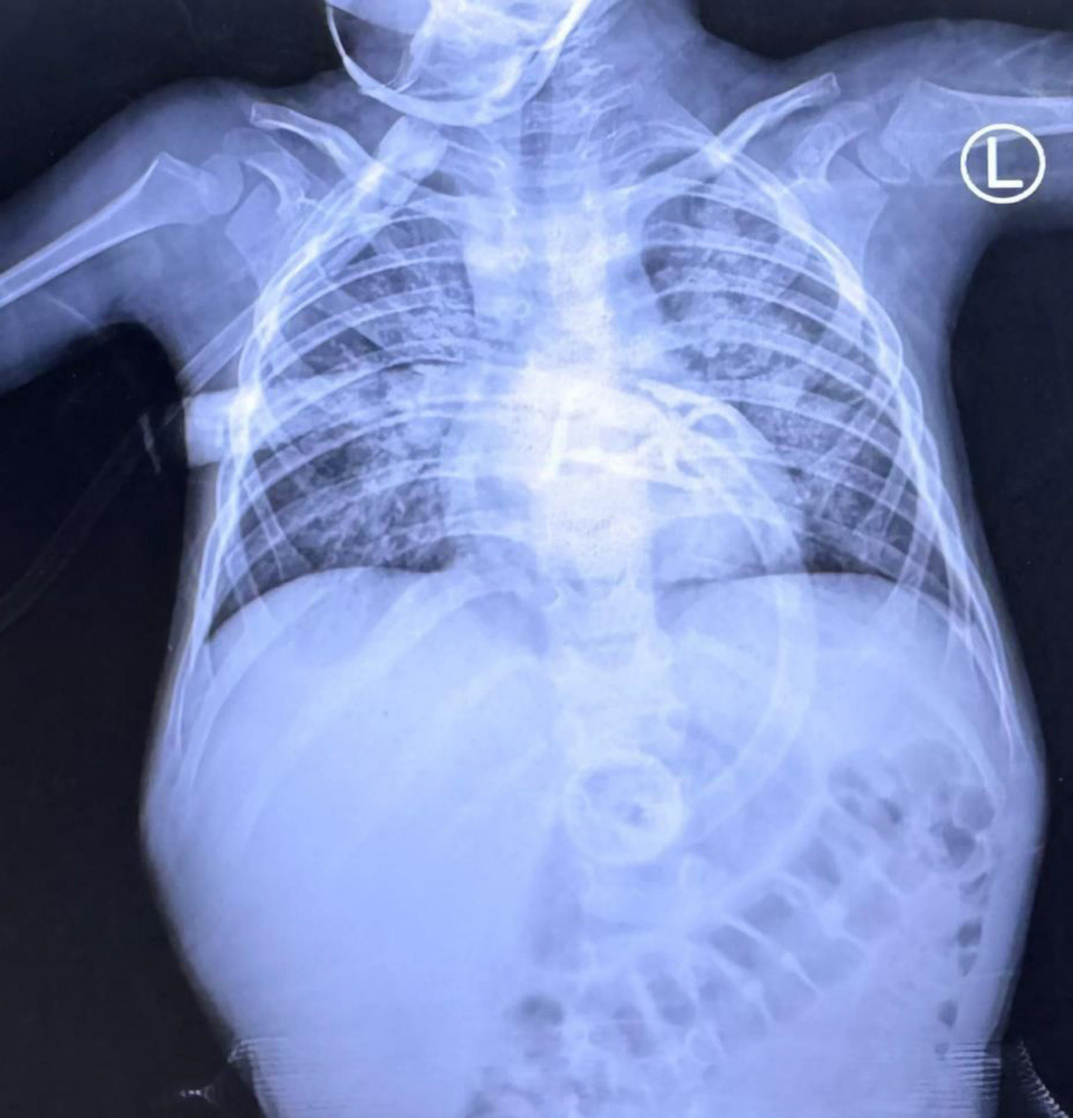
Chest radiograph obtained on the day of admission, demonstrating bilateral interstitial infiltrates with prominent perihilar markings, consistent with viral pneumonitis. The image was taken shortly after PICU admission as part of the initial diagnostic workup.

A chest radiograph (Figure 1) revealed diffuse bilateral interstitial infiltrates with prominent perihilar markings, consistent with a viral pneumonitis pattern. No focal consolidation, pleural effusion, or pneumothorax was identified. The cardiac silhouette was normal in size, and the mediastinum appeared unremarkable. The diaphragms—particularly on the right side—were mildly elevated, possibly reflecting decreased lung volumes or abdominal distension. Abdominal gas patterns were visible, consistent with gastrointestinal dysmotility or ileus, a known complication in critically ill pediatric patients. A gastrostomy tube was noted in situ with appropriate positioning. Overall, these radiographic findings aligned with the patient’s clinical presentation of H1N1 influenza-induced respiratory distress and supported the diagnosis of viral pneumonia in the setting of HLH.

The patient tested positive for H1N1 influenza and was initiated on oseltamivir (3 mg/kg/dose twice daily for five days) while continuing vancomycin therapy. Two days after testing positive, the patient experienced convulsions accompanied by a decline in consciousness, as well as episodes of abnormal laughter and paroxysmal crying. New-onset paroxysmal crying and abnormal laughter, together with EEG-confirmed ictal activity, suggested central nervous system (CNS) involvement by HLH, distinct from the patient’s baseline seizure disorder. Management included Ventolin (albuterol) 0.15 mg/kg every 4 hours, hypertonic saline, and nebulized budesonide (Pulmicort) 0.25–0.5 mg twice daily. An echocardiogram performed at this time was unremarkable. Following 14 days of therapy, the patient achieved remission, with resolution of fever, normalization of inflammatory markers (ferritin: 250 ng/mL), and stabilization of hematologic parameters (platelets: 220×10^9^/L). The patient remained hospitalized for a total of 21 days, including 10 days in the pediatric intensive care unit, followed by four days in the general pediatric ward. He was discharged home in stable condition on maintenance dexamethasone, without the need for supplemental oxygen. A follow-up plan was arranged through the hospital’s pediatric immunology and neurology clinics, with the first follow-up visit scheduled two weeks post-discharge, and a subsequent evaluation at three months. At follow-up, he remained clinically well, with no recurrence of HLH symptoms or seizure activity.

## 3. DISCUSSION

Hemophagocytic lymphohistiocytosis (HLH) is a life-threatening hyperinflammatory syndrome caused by dysregulated immune activation, leading to persistent, excessive inflammation that affects multiple organ systems.^6^ HLH is classified as either primary (familial), resulting from genetic defects in cytotoxic T cells and natural killer (NK) cells, or secondary, which arises in response to an underlying trigger.^7^ Primary HLH was ruled out in this patient due to the absence of a personal or family history of immunodeficiency, a finding further confirmed by genome sequencing, which showed no HLH-related mutations. The presence of an acute viral infection strongly supported a diagnosis of secondary HLH.

Unlike primary HLH, which is driven by genetic mutations, secondary HLH results from immune dysregulation triggered by infections, malignancies, autoimmune disorders, or inflammatory conditions.^8^ It is most commonly associated with viral infections, particularly Epstein–Barr virus (EBV), cytomegalovirus (CMV), herpes simplex virus (HSV), and human immunodeficiency virus (HIV), but can also occur in the context of bacterial (e.g., *Mycobacterium tuberculosis*), fungal (e.g., *Candida*), and parasitic (e.g., *Leishmania*) infections.^9^ This patient met five of the eight HLH-2004 diagnostic criteria, with the confirmed H1N1 influenza infection likely serving as the trigger factor for secondary HLH.

H1N1 is a recognized HLH trigger, particularly in immunocompromised individuals.^10^ In patients with DS, respiratory infections can escalate to systemic hyperinflammation due to impaired viral clearance and dysregulated cytokine responses. H1N1 is primarily a respiratory pathogen that infects swine, with zoonotic transmission to humans occurring through direct or indirect contact with infected animals. In this case, the patient’s respiratory symptoms were consistent with H1N1 infection, which is particularly concerning given the immunological vulnerabilities associated with DS. Individuals with DS exhibit impairments in both innate and adaptive immunity, including defective neutrophil chemotaxis and diminished humoral responses, which predispose them to recurrent respiratory infections. Additionally, zinc deficiency and premature immunosenescence have been associated with immune dysfunction in DS, though their precise clinical significance remains unclear.^11^ Structural anomalies, including airway malformations and hypotonia, further impair mucociliary clearance and protective barriers, increasing the susceptibility to respiratory pathogens and highlighting the need for tailored management strategies in patients with DS.^12^ The patient’s neurological deterioration, marked by seizures, episodic abnormal laughter, and crying spells, may reflect the neurologic involvement of HLH, which occurs in approximately two-thirds of cases and is associated with poorer prognostic outcomes.^13^ Additionally, this case highlights the value of home pulse oximetry monitoring in medically complex pediatric patients. The parents’ ability to detect sustained hypoxemia through home SpO_2_ monitoring facilitated early medical evaluation, which was crucial for the timely diagnosis and management of this rapidly evolving condition.

To differentiate primary (familial) HLH from secondary causes,^14^ targeted genomic sequencing was performed using a panel of genes associated with primary HLH, including *PRF1, UNC13D, STXBP2*, and *XIAP*. No pathogenic variants were identified, effectively excluding genetic etiology for HLH. This distinction is clinically significant, as primary HLH typically requires hematopoietic stem cell transplantation for cure,^15^ whereas secondary HLH can often be managed with immunosuppression and treatment of the triggering condition.

The management of HLH involves addressing the underlying trigger, providing supportive care, and administering immunosuppressive and anti-inflammatory therapy to control the hyperinflammatory response and prevent immune-mediated organ damage. Standard treatment regimens, as outlined in the HLH-94 and HLH-2004 protocols, include high-dose corticosteroids (e.g., dexamethasone 10 mg/m^2^), cytotoxic agents (etoposide), immunosuppressants (cyclosporin A), immunomodulators (intravenous immunoglobulin), monoclonal antibodies (rituximab, alemtuzumab), and anti-cytokine agents (anakinra, thymoglobulin).^7,16^ In this case, corticosteroid and etoposide therapy effectively controlled the patient’s hyperinflammatory response without adverse effects, while antiviral treatment with oseltamivir targeted the underlying H1N1 infection, thereby eliminating the need for additional immunosuppressive agents.

Due to several key factors, this case adds a new dimension to the literature on HLH in DS. Unlike previously reported cases, which primarily involved infectious triggers such as SARS-CoV-2, *Mycoplasma pneumoniae*,^17,18^ and other opportunistic pathogens, this report identifies H1N1 influenza as a trigger for secondary HLH in an infant with DS. Patients with DS are also predisposed to hematologic malignancies, requiring treatment strategies that balance effective HLH control with minimizing toxicity. Despite this, standard full-dose HLH therapy, including corticosteroids and etoposide, was successfully administered without complications, challenging the commonly held belief that etoposide poses an unacceptable risk in immunocompromised patients or those with DS.^19^ Furthermore, unlike prior reports that employed targeted interferon inhibition (emapalumab, baricitinib)^20,21^ as a steroid-sparing strategy, this case demonstrates that HLH in DS can be effectively managed with conventional immunosuppressive therapy alone, without the need for novel biologic agents. The resolution of HLH without recurrence suggests that interferon inhibition may not be necessary in all cases, particularly when HLH is driven by acute infection rather than chronic immune dysregulation.

## 4. CONCLUSION

This case illustrates the rarity and complexity of secondary HLH in a pediatric patient with trisomy 21, exacerbated by an H1N1 influenza infection. This case highlights the rarity and clinical challenge of secondary HLH in an infant with DS triggered by H1N1 infection. The successful use of standard HLH therapy, including corticosteroids and etoposide, in this vulnerable population highlights the importance of individualized treatment. The favorable outcome challenges previous concerns regarding immunosuppressive toxicity in DS. Further research is needed to establish standardized management protocols for such cases. This report adds to the growing body of literature on HLH in DS and emphasizes the urgent need for further research to establish clear, evidence-based treatment recommendations for this uniquely vulnerable patient population.

## LIST OF ABBREVIATIONS

**Table:** 

ANC	Absolute Neutrophil Count
CNS	Central Nervous System
DS	Down Syndrome
EEG	Electroencephalogram
GERD	Gastroesophageal Reflux Disease
HLH	Hemophagocytic Lymphohistiocytosis

## COMPETING INTERESTS

The authors have no conflicts of interest to declare.

## Figures and Tables

**Table 1.** Comparison of HLH-2004 diagnostic criteria with the patient’s clinical and laboratory findings.

HLH-2004 diagnostic criteria	Patient’s findings	Met	Reference range
1. Fever	Present (persistent fever)	Yes	Temperature ≥38.5°C
2. Splenomegaly	Splenomegaly noted on examination	Yes	Clinical/palpable/imaging-confirmed
3. Cytopenias (≥2 lineages):		Yes	
- Hemoglobin	10.1 g/dL	↓	>11.2 g/dL (age-adjusted)
- Platelets	136×10^9^/L	↓	>150×10^9^/L
- Neutrophils (ANC)	130 cells/μL	↓	>1,500 cells/μL
4. Hyperferritinemia	>1,650 ng/mL	Yes	<200 ng/mL (lab-specific)
5. Hypofibrinogenemia	101 mg/dL	Yes	160–400 mg/dL
6. Hemophagocytosis	Not assessed (bone marrow biopsy not performed)	No	Bone marrow/spleen/LN (Lymph Node) biopsy
7. Elevated sCD25 (soluble Interleukin-2 receptor)	Not tested	N/A	>2,400 U/mL (age-adjusted)
8. Impaired NK-cell activity	Not tested	N/A	Functional assay required
9. Hypertriglyceridemia	Not tested	N/A	Fasting >265 mg/dL
Genetic mutations (primary HLH)	Negative (no *PRF1/UNC13D/STXBP2/XLP*)	No	N/A

## References

[B1] Bull MJ (2020;). Down syndrome. N Engl J Med.

[B2] Kusters MA, Verstegen RH, Gemen EF, de Vries E (2009;). Intrinsic defect of the immune system in children with Down syndrome: A review. Clin Exp Immunol.

[B3] Janka GE (2007;). Hemophagocytic syndromes. Blood Rev.

[B4] Ramos-Casals M, Brito-Zerón P, López-Guillermo A, Khamashta MA, Bosch X (2014;). Adult haemophagocytic syndrome. Lancet.

[B5] Hainsworth JD, Greco FA (1995;). Etoposide: Twenty years later. Ann Oncol.

[B6] Wu Y, Sun X, Kang K, Yang Y, Li H, Zhao A (2024;). Hemophagocytic lymphohistiocytosis: Current treatment advances, emerging targeted therapy and underlying mechanisms. J Hematol Oncol.

[B7] George MR (2014). Hemophagocytic lymphohistiocytosis: Review of etiologies and management. J Blood Med.

[B8] Hameed M, Garcia G, Zhen KC, Hartenstein B, Herrera LDJ, Hamad K (2024;). Secondary hemophagocytic lymphohistiocytosis (HLH): A systematic review of incidence and prognostic outcomes. Blood.

[B9] Allen CE, McClain KL (2015;). Pathophysiology and epidemiology of hemophagocytic lymphohistiocytosis. Hematology Am Soc Hematol Educ Program.

[B10] Lai S, Merritt BY, Chen L, Zhou X, Green LK (2012;). Hemophagocytic lymphohistiocytosis associated with influenza A (H1N1) infection in a patient with chronic lymphocytic leukemia: An autopsy case report and review of the literature. Ann Diagn Pathol.

[B11] Jilani TN, Jamil RT, Nguyen AD, Siddiqui AH (2025). H1N1 influenza. StatPearls.

[B12] Ram G, Chinen J (2011;). Infections and immunodeficiency in Down syndrome. Clin Exp Immunol.

[B13] Horne A, Wickström R, Jordan MB, Yeh EA, Naqvi A, Henter JI (2017;). How to treat involvement of the central nervous system in hemophagocytic lymphohistiocytosis?. Curr Treat Options Neurol.

[B14] Canna SW, Marsh RA (2020;). Pediatric hemophagocytic lymphohistiocytosis. Blood.

[B15] Kim H, Mizuno K, Masuda K, Sakurai M, Ara T, Naito K (2024;). A nationwide retrospective analysis of allogeneic hematopoietic stem cell transplantation for adult hemophagocytic lymphohistiocytosis. Transplant Cell Ther.

[B16] Henter JI, Horne A, Aricó M, Egeler RM, Filipovich AH, Imashuku S (2007;). HLH-2004: Diagnostic and therapeutic guidelines for hemophagocytic lymphohistiocytosis. Pediatr Blood Cancer.

[B17] Lazea C, Blag C (2018;). Hemophagocytic lymphohistiocytosis secondary to Mycoplasma pneumoniae infection in a trisomy 21 girl. Rev Rom Med Lab.

[B18] Kim-Hellmuth S, Hermann M, Eilenberger J, Ley-Zaporozhan J, Fischer M, Hauck F (2021). SARS-CoV-2 triggering severe acute respiratory distress syndrome and secondary hemophagocytic lymphohistiocytosis in a 3-year-old child with Down syndrome. J Pediatric Infect Dis Soc.

[B19] Dupont T, Darmon M, Mariotte E, Lemiale V, Fadlallah J, Mirouse A (2022;). Etoposide treatment in secondary hemophagocytic syndrome: Impact on healthcare-associated infections and survival. Ann Intensive Care.

[B20] Agress A, Malle L, Eisenberg R, Bogunovic D (2022;). Five interferon receptor triplicates: A unique case of hemophagocytic lymphohistiocytosis in a Down syndrome patient. Ann Allergy Asthma Immunol.

[B21] Guild A, Fritch J, Patel S, Reinhardt A, Acquazzino M (2022). Hemophagocytic lymphohistocytosis in trisomy 21: Successful treatment with interferon inhibition. Pediatr Rheumatol Online J.

